# Identification of an Internal RNA Element Essential for Replication and Translational Enhancement of *Tobacco Necrosis Virus A* ^C^

**DOI:** 10.1371/journal.pone.0057938

**Published:** 2013-02-27

**Authors:** Heng Pu, Jiang Li, Dawei Li, Chenggui Han, Jialin Yu

**Affiliations:** State Key Laboratories for Agrobiotechnology, College of Biological Sciences, China Agricultural University, Beijing, China; Nanjing Agricultural University, China

## Abstract

Different regulatory elements function are involved in plant virus gene expression and replication by long-distance RNA-RNA interactions. A cap-independent functional element of the *Barley yellow dwarf virus* (BYDV) – like translational enhancer (BTE) is present in *Tobacco necrosis virus A* (TNV-A), a *Necrovirus* member in the *Tombusviridae* family. In this paper, an RNA stretch flanking the 5′ proximal end of the TNV-A^C^ coat protein (CP) gene was shown to be essential for viral replication in *Chenopodium amaranticolor* plants and tobacco cells. This internal sequence functioned in transient expression of β-glucuronidase (GUS) when present at either the 5′ or 3′ sides of the GUS open reading frame. Serial deletion analyses revealed that nine nucleotides from nt 2609 to 2617 (−3 to +6 of the CP initiation site) within TNV-A^C^ RNA are indispensable for viral replication in whole plants and tobacco cells. Fusion of this RNA element in mRNAs translated in tobacco cells resulted in a remarkable enhancement of luciferase expression from *in vitro* synthesised chimaeric RNAs or DNA expression vectors. Interestingly, the element also exhibited increased translational activity when fused downstream of the reporter genes, although the efficiency was lower than with upstream fusions. These results provide evidence that an internal RNA element in the genomic (g) RNA of TNV-A^C^, ranging approximately from nt 2543 to 2617, plays a bifunctional role in viral replication and translation enhancement during infection, and that this element may use novel strategies differing from those previously reported for other viruses.

## Introduction

Positive-stranded RNA viruses often harbour RNA elements within their genomic (g) RNAs that mediate a variety of fundamental viral processes. Viral RNA elements were conventionally viewed as localised sequences or structures, such as poly(A) tails, pseudoknots, tRNA-like structures or RNA hairpins [1]. However, in recent years, compelling evidence has revealed that such RNA elements may function via long-distance interactions to control viral translation, replication and transcription [2]. For instance, long-distance base-pairing communications between the 5′ and 3′ untranslated regions (UTRs) of BYDV [3] or *Tomato bushy stunt virus* (TBSV) [4] gRNAs facilitate cap-independent translation. Moreover, long-range interactions between internal gRNA segments are required for initiation of subgenomic (sg) mRNA transcription in various plant viruses, including *Red clover necrotic mosaic virus* (*Dianthovirus*) [5], *Cucumber leaf spot virus* (*Aureusvirus*) [6], *Potato virus X* (*Potexvirus*) [7] and TBSV [8]–[10]. These results demonstrate the prevalence and fundamental importance of regulatory RNA elements for diverse reproductive strategies. In the uncapped [11] and nonpolyadenylated [12]–[14] *Necrovirus* gRNAs, cap-independent functional elements related to the BYDV–like translational enhancer (BTE) in the 3′ UTR are involved in long-distance RNA-based interactions during translation and replication. These interactions have been well studied to understand the mechanistic features of virus reproduction [15]–[17], in which the sgRNA2 leader of TNV-A can interact synergistically with the BYDV 3′ element to promote *in vitro* translation [17].

TNV-A is the type member of the *Necrovirus* genus in the *Tombusviridae* family [18] and the virus causes damage to many economic crops. A TNV-A isolate, designated TNV-A^C^ that was obtained from soybean (*Glycine max*) in China, causes a localised infection in *Chenopodium amaranticolor* leaves and systemic infections in soybean and *Nicotiana benthamiana* [19]. TNV-A^C^ has a 3682 nucleotide (nt) linear monopartite gRNA [20], and shares most features of its genomic organisation and expression strategy with other necroviruses [14], [21]. In these viruses, three proteins are encoded by two sg mRNAs transcribed from the 3′-proximal end of the gRNA [13], [17], [21], [22]. These include the 8 kD (P8), 6 kD (P6) and 30 kD (coat protein) proteins required for cell-to-cell movement, and/or long-distance systemic infection [23].

We reported previously that the intact TNV-A^C^ coat protein (CP) is dispensable for infection of *C. amaranticolor*, and that the 5′ terminal nucleotides (NT) of the CP coding region affects local symptoms and viral RNA accumulation [22]. These results thus demonstrate an important role for the internal sequence in regulation of viral replication and symptom severity. To clarify the mechanisms of RNA-based regulation by the TNV-A^C^ element, we have now precisely identified functional nucleotides flanking the CP initiation site by site-directed mutagenesis. We also demonstrated pronounced positional effects of an internal element on viral RNA synthesis and translational enhancement of a reporter gene fused to the element.

## Results

### Functional analysis of the coat protein gene by internal fragment deletions

**Figure 1:**
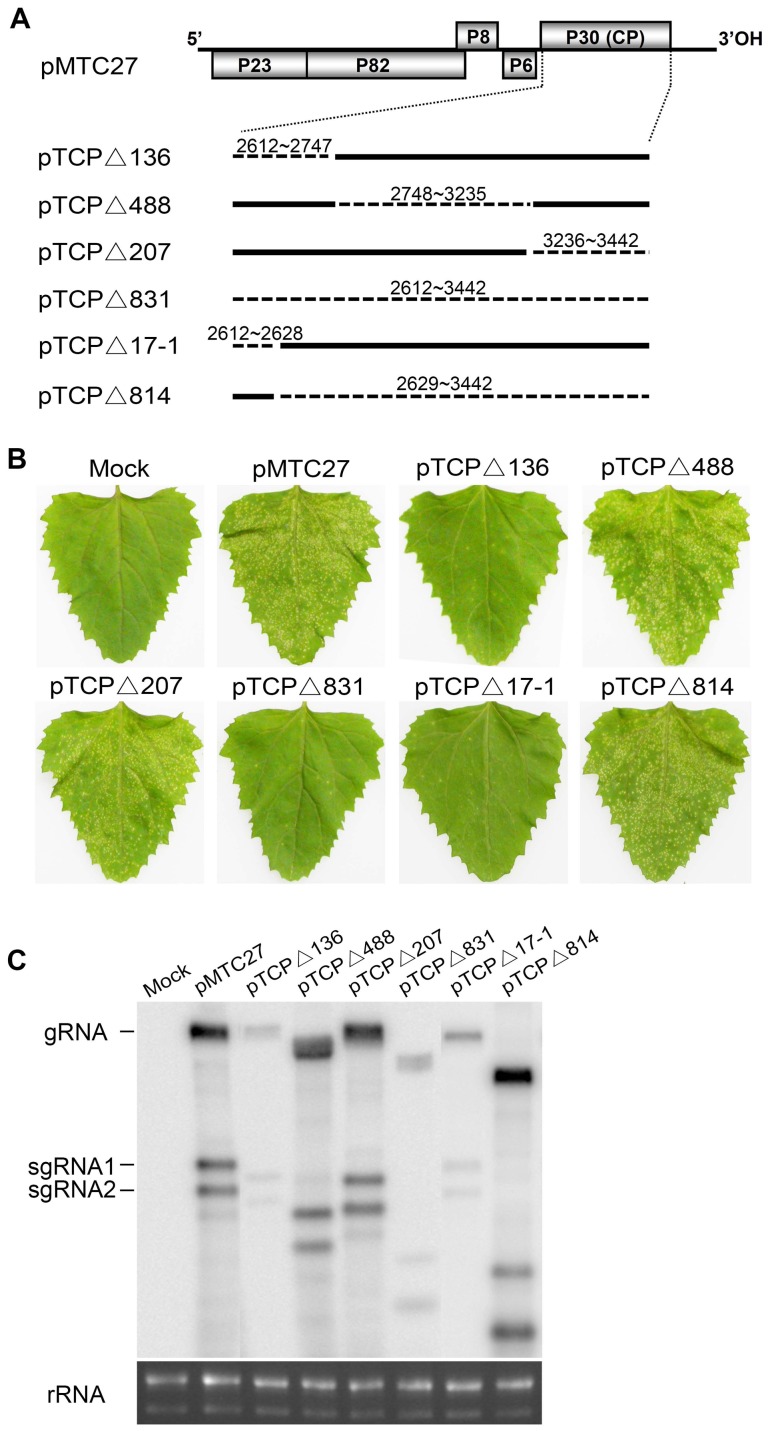
Deletions of the TNV-A^C^ coat protein gene and plant inoculations. (A) Genomic organization of wtTNV-A^C^ (pMTC27) ORFs are shown as gray boxes. The six mutant constructs are shown below and the associated dashed lines indicate deleted nucleotide fragments whose positions within TNV RNA are identified by numbers within the deleted regions. (B) The phenotype of *C. amaranticolor* leaves inoculated with mutant RNAs were photographed at 4 dpi. Viral RNAs used for inoculations are shown above each photo, including a mock inoculation with buffer alone on the upper left leaf. (C) Northern blot detection of wtTNV-A^C^ and deletion mutants viral RNAs isolated from inoculated *C. amaranticolor* leaves. A cDNA fragment derived from the TNV-A^C^ 3′ UTR was labelled with a ^32^P labeled probe to assess viral RNA accumulation. The positions of TNV-A^C^ RNA species are indicated on the left side of the gel photographs, and plant ribosomal RNAs (rRNA) used as loading controls are shown in the bottom panel.

Our previous results revealed that abolishing expression of the TNV-A^C^ CP by either deleting the entire coding region or by prematurely terminating the translational start codon with a CAC triplet resulted in attenuated local symptoms and reduced levels of viral RNA accumulation in *C. amaranticolor* plants [22]. To determine whether the symptom severity and the viral RNA replication were modulated by the intact CP or by a functional RNA element in the CP coding region, we constructed a series of mutants (Fig. 1A, S1A) and tested these by mechanical inoculation of *C. amaranticolor*. At 4 dpi, the results revealed that deletions in the 5′ end of the coding region (pTCPΔ136, pTCPΔ831 and pTCPΔ17-1) eliminated symptoms and greatly reduced RNA synthesis, compared to the internal or the 3′ end deletions TNV-A^C^ gRNA (pTCPΔ488, pTCPΔ207, pTCPΔ814 and other mutants derived from pTCPΔ136), which had elevated RNA levels similar to those of wild type (wt) TNV-A^C^ (Fig. 1B and C, S1B and S1C). Among these mutants, only pTCPΔ207 contained an in-frame deletion, and the CP subunits expressed from this mutant were smaller than the wt TNV-A^C^ CP (data not shown). These results suggest the existence of a gRNA element near the 5′-proximal end of the CP gene that is responsible for viral RNA synthesis. A mutant containing this element is represented by pTCPΔ814, which contains the 17 nt at the 5′ end of the CP gene, but lacks the remainder of the CP ORF and the stop codon (Fig. 1).

**Figure 2:**
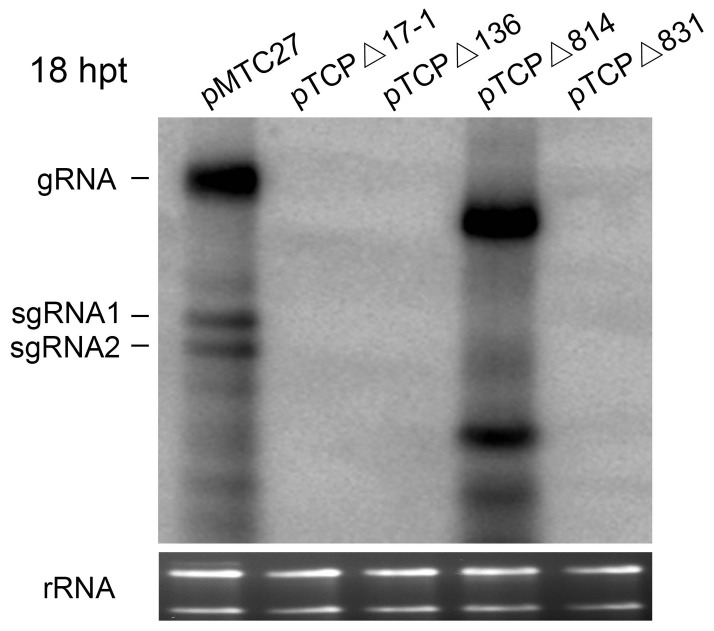
Accumulation of TNV-A^C^ RNAs in tobacco BY-2 protoplasts transfected with viral mutant RNAs. Total RNA was isolated from inoculated BY-2 protoplasts at 18 hpt. Mutants used for inoculation are illustrated above each lane. Viral RNA accumulation was assessed by Northern blots with the same probe used in Fig. 1, and designations along the side of the gel are similar to those in Fig. 1.

In addition, introduction of pTCPΔ814, pTCPΔ17-1, pTCPΔ136 and pTCPΔ831 into BY-2 protoplasts showed that viral RNAs accumulated only in cells transfected by pTCPΔ814 or wt TNV-A^C^ constructs, both of which retained the 17-nt CP fragment, but protoplasts infected with the other three constructs failed to accumulate substantial amounts of viral RNAs (Fig. 2). These results confirm that the first 17-nt of TNV-A^C^ CP gene is indispensable for high levels of viral RNA replication in *C. amaranticolor*, although very low levels of mutated RNAs lacking the 17-nt region could be found in plant tissues.

### Test of the putative enhancement element by GUS expression

**Figure 3:**
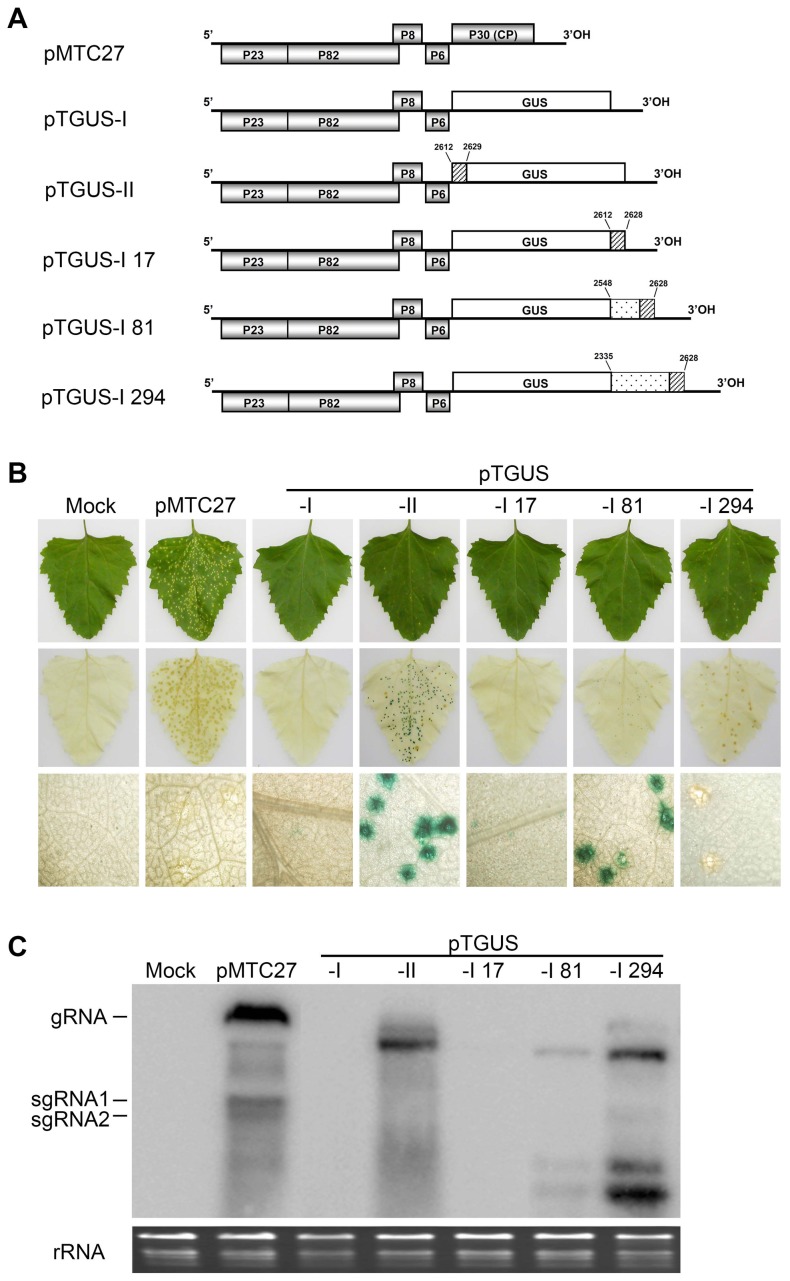
GUS expression of the TNV-A^C^ recombinants. (A) Schematic illustration of wtTNV-A^C^ (pMTC 27), pTGUS-I and the recombinant derivatives pTGUS-II, pTGUS-I 17, pTGUS-I 81 and pTGUS-I 294. TNV ORFs are shown as gray boxes, the GUS gene as a white box, the 5′ proximal ends of the CP gene (nt 2612–2629 or nt 2612–2628) are illustrated as a box with diagonal lines, and the duplicate sequences upstream of the CP gene are represented by dotted boxes. The numbers identify the locations of the inserted nucleotide positions in TNV gRNA. (B) Inoculated *C. amaranticolor* leaf phenotypes were photographed at 4 dpi, and viral RNAs used for inoculations are shown above each photo (Top panel). The results of GUS histochemical staining are shown without magnification (Middle panel) or under a light microscope at 40X magnification (Bottom panel). The inoculated leaves were stained with 1 mM X-Gluc in 50 mM NaH_2_PO_4_ (pH 7.0) and incubated at 37°C in darkness overnight. (C) Viral RNA accumulation was assessed by Northern blots with the same probe used in Fig. 1. The migration of viral RNAs and intensities of the rRNA loading control species are as indicated in Fig. 1.

Because the 17-nt sequence is located at the 5′ proximal end of the TNV-A^C^ CP gene, it is possible that a more complete replicative element might consist of additional upstream nucleotides. To test this possibility, a β-glucuronidase (GUS) reporter was substituted into the TNV-A^C^ genome to replace the CP gene (nt 2612–3442) to produce pTGUS-I, or was fused in-frame with the 5′ proximal 18 nucleotides (nt 2612–2629) of the CP gene to generate pTGUS-II (Fig. 3A). In addition, three other recombinants based on pTGUS-I were created for GUS expression at the 3′ proximal end of the GUS ORF (Fig. 3A). The plasmid pTGUS-I 17 consisted of an ectopic 3′ fusion of the 17-nt element (nt 2612–2628), pTGUS-I 81 contained an additional 64 residues (nt 2548–2611) 3′ fusion and pTGUS-I 294 had 277 residues (nt 2335–2611) fusion (Fig. 3A). After inoculation with recombinant virus transcripts, localised symptoms and viral RNA accumulation were evaluated in *C. amaranticolor* at 4 dpi. In contrast to the necrotic lesions caused by wt TNV-A^C^ (pMTC27), attenuated symptoms consisting of a few chlorotic spots were observed in leaves inoculated with pTGUS-II and pTGUS-I 294, whereas lesions failed to appear on leaves inoculated with pTGUS-I, pTGUS-I 17 and pTGUS-I 81 (Fig. 3B, top panel). Histochemical staining [24], [25] revealed many blue GUS lesions in leaves inoculated with pTGUS-II, whereas fewer lesions were evident after inoculation with pTGUS-I 81 (Fig. 3B, middle panel). In contrast, on leaves inoculated with pTGUS-I 17, very faint blue staining could be seen only at higher magnification (40X) with a IX71® Inverted Microscope (Olympus); no staining was seen in leaves inoculated with pTGUS-I or pTGUS-I 294 (Fig. 3B, bottom panel). These results suggest that the viral element is length dependent, that an appropriate size is required for high levels of GUS expression in the leaves (Fig. 3B, middle and bottom panels), and that the element has positional effects that permit functioning either at the 5′ or 3′ end of the ORF. Consistent with the symptom severity, Northern blot analysis showed that the levels of viral RNA accumulation in leaves inoculated with pTGUS-II and pTGUS-I 294 were substantially higher than for pTGUS-I 81. However, a trace amount of RNA was present following pTGUS-I 17 inoculation, but no detectable viral RNA could be detected after pTGUS-I inoculation (Fig. 3C), nor were GUS sgRNAs in the leaves inoculated with the GUS recombinants when a specific probe was used in a parallel experiment (data not shown). Compared to wt TNV-A^C^, smaller sized viral RNA bands were present in plants infected by the GUS recombinants (Fig. 3C). By sequencing of three cDNA clones derived from each pTGUS construct, spontaneous deletions of GUS reporters from the recombinant viruses during replication were evaluated and the recombination sites in the progeny viruses were identified (data not shown). In cases where viral RNA fragments were fused downstream of the GUS reporter gene (pTGUS-I 17, pTGUS-I 81 and pTGUS-I 294), the levels of the shorter sized viral RNAs were positively correlated with the length of the fused viral sequences, possibly reflecting the frequency of homologous recombination events resulting in rescue of the functional requirements necessary for viral replication. From these results, we suggest that a functional element necessary for transient gene expression is present in the internal sequence upstream of the 17-nt region of TNV-A^C^ CP gene, and the results indicate that this element can function independently of its 5′ or 3′ position around the GUS reporter ORF. Since no protein coding capability is predicted in the sequence, we conclude that a non-coding RNA element in the central region of the TNV-A^C^ genome functions in viral replication and translation.

### Analysis of the RNA element by nucleotide deletions

**Figure 4:**
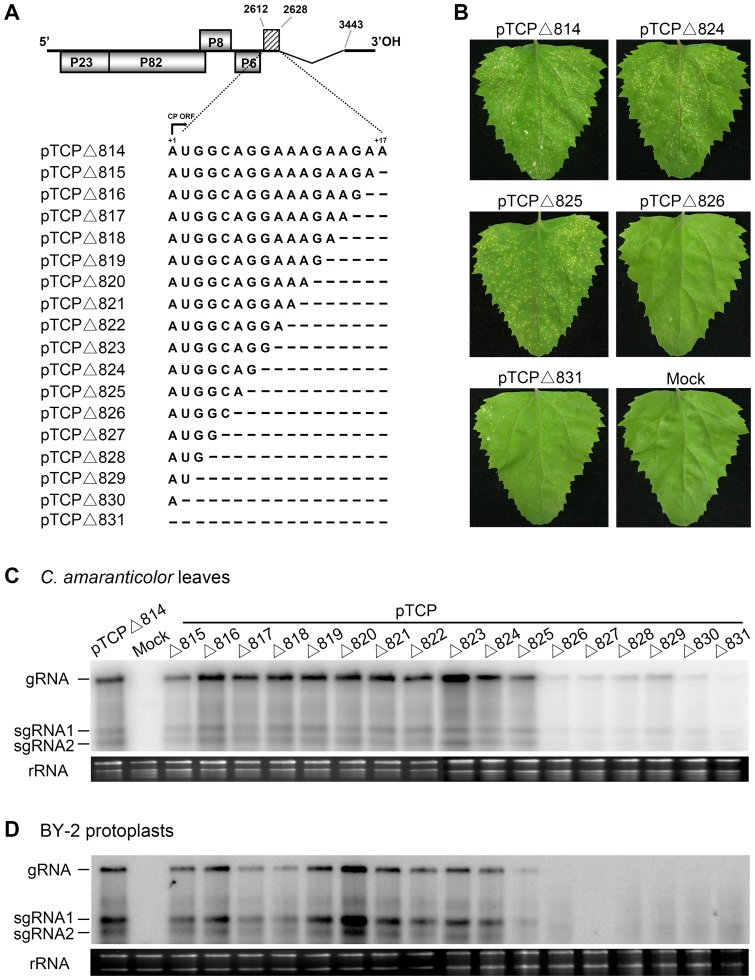
Deletion mapping of the 17-nt region within the coat protein. (A) Schematic illustration pTCPΔ814 is shown on the top and the angular lines represent the deleted 3′ region of the CP ORF (nt 2629–3442). The diagonal lines indicate the 17 nt sequence at 5′ end of the CP gene that was subjected to deletion analysis. Sequentially deleted nucleotides in the 17-nt region (nt 2612–2628), encompassing +1 to +17 of the translation initiation site are denoted by dashed lines. (B) *C. amaranticolor* leaf phenotypes elicited by the mutants (pTCPΔ814, pTCPΔ824, pTCPΔ825, pTCPΔ826 and pTCPΔ831) were photographed at 4 dpi, and the mutant viral RNA derivatives used for inoculations are illustrated above each photo. Viral RNA accumulation in *C. amaranticolor* plants (C) or tobacco cells (D) was assessed by Northern blots with the same probe used in Fig. 1. The TNV-A^C^ RNA species and plant rRNA loading controls are indicated in panels of C and D.

To map the 3′ boundaries of the RNA element, 16 constructs were made by single base deletions starting from the 3′ end of the 17-nt region in pTCPΔ814 (Fig. 4A). After mechanical inoculation of *C. amaranticolor* with the resulting mutant viral RNA *in vitro* transcripts, local lesion symptoms similar to those elicited by pTCPΔ814 appeared on leaves inoculated with 11 of the 16 constructs. These include pTCPΔ815 to pTCPΔ825, each of which retain at least 6 nts at the 5′ end of the 17-nt region, whereas the same lack of symptoms seen with pTCPΔ831 inoculation occurred with the other five mutants, pTCPΔ826 to pTCPΔ830, which harbor 1 to 5 nts at 5′ end of the CP ORF (Fig. 4B). Northern blot analysis revealed relatively high levels of viral RNAs in the *C. amaranticolor* leaves inoculated with RNAs of the first 11 constructs (pTCPΔ815 to pTCPΔ825), but much lower levels were present in leaves inoculated with pTCPΔ826 to pTCPΔ830 RNAs (Fig. 4C). Consistent results were obtained in BY-2 cells, where viral RNAs were barely detectable in cells transfected with the pTCPΔ826 to pTCPΔ830 RNAs (Fig. 4D). These analyses demonstrated that the 3′ terminus of the RNA element is located at position 2617 at the 5′ end of the CP gene.

**Figure 5:**
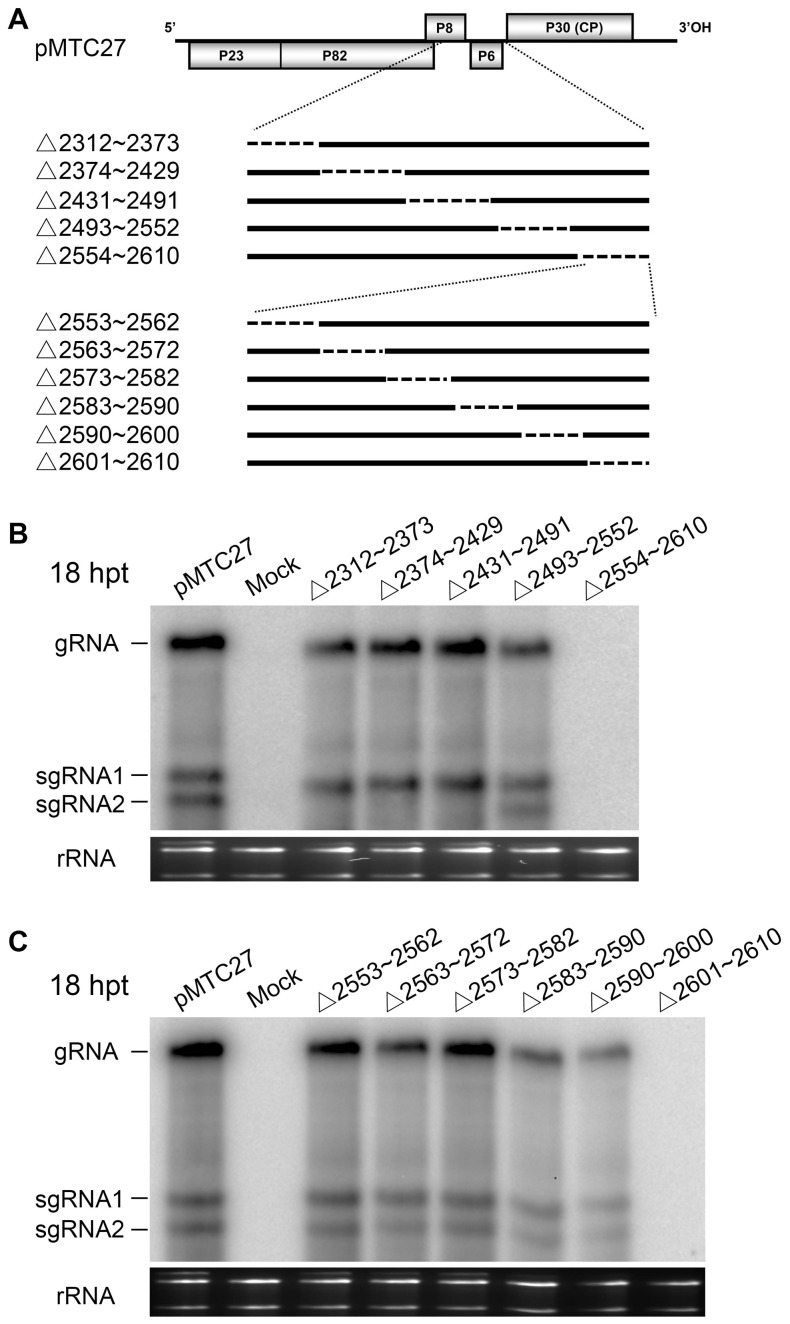
Deletion analysis of the sequence upstream of the coat protein gene and detection of viral RNAs in protoplasts. (A) The wtTNV-A^C^ (pMTC 27) construct is shown on top, and the two regions (nt 2312–2610 and nt 2553–2610) upstream of the CP gene that were subjected to deletion are expanded below, and the deleted nucleotides are indicated by dashed lines. Viral RNA accumulation in tobacco BY-2 protoplasts transfected by RNAs containing deletions within the nt 2312–2610 region (B) or the nt 2553–2610 region (C) were assessed by Northern blots at 18 hpt with the same probe used in Fig. 1. Illustration of the viral RNAs and rRNA species are indicated on the left side of the gel photographs in the panels C and D.

To identify the 5′-proximal nucleotide of the functional element, five mutants were constructed by truncation of approximately 60 nts from the internal region of pMTC27. This region is flanked by nt 2311 in the middle of the p8 gene and nt 2611 immediately adjacent to the CP translational initiation codon (Fig. 5A). Northern blot analysis of transfected BY-2 protoplasts indicated that high levels of viral RNA were present in cells transfected with constructs that were most similar to wt TNV-A^C^, whereas no accumulation was detected in cells transfected with Δ2554–2610 RNAs (Fig. 5B). Approximately 10 nts were progressively deleted from each of six constructs flanking the 2553 to 2610 nt region in pMTC27 (Fig. 5A). Viral RNA was undetectable by Northern blot analysis in cells transfected with Δ2601–2610, and reduced levels of RNA were present in Δ2583–2590 or Δ2590–2600 transfected cells, in contrast to the wt TNV-A^C^-like levels evident in cells with Δ2553–2562, Δ2563–2572 and Δ2573–2582 RNAs (Fig. 5C). The responses to transfection with the series of mutant plasmids described above indicate that nucleotides downstream of nt 2601 are part of the region that is essential for viral replication, while the flanking sequence up to nt 2583 may contribute to highly efficient replication, as is consistent with the results shown in Fig. S2.

**Figure 6:**
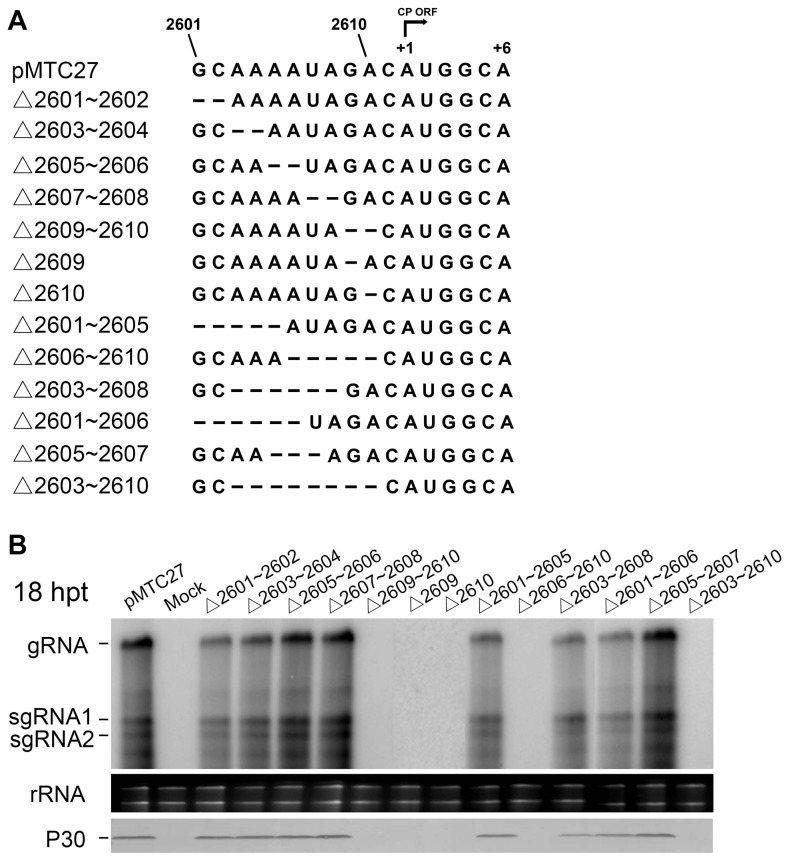
Single and multiple nucleotide deletions within the 17 nt functional region and detection of viral RNAs in protoplasts. (A) The 17 nt functional region (nt 2601–2617) of wtTNV-A^C^ (pMTC 27) is shown on top, and the arrow indicates the translation direction from the +1 start site of the CP gene to the +6 position. Nucleotides deleted in the mutants are indicated by dashed lines. (B) Viral RNA accumulation in tobacco BY-2 protoplasts transfected by the mutants was assessed by Northern blots at 18 hpt with the same probe used in Fig. 1 and the CP (P30) was identified in Western blots with a antibody raised against the TNV-A^C^ CP. The TNV-A^C^ RNA species and CP (P30) are indicated on the left side of the gel photographs and plant rRNAs are shown as loading controls in the bottom panels.

To precisely map the 5′ boundary of the regulatory element, a series of mutants were constructed from pMTC27 by deletion of adjacent nucleotides within the 2601 to 2610 nt region (Fig. 6A). Molecular analysis showed that viral RNA and CP accumulation was barely detectable in the cells transfected by the mutants lacking either nt 2609 or nt 2610, in contrast to high levels of replication in cells transfected by other mutants that retain both nt 2609 and 2610 (Fig. 6B). These data demonstrate that the internal RNA regulatory element essential for TNV-A^C^ amplification is composed of 9 nucleotides from nt 2609 to 2617, and is located at positions −3 to +6 of the CP translation initiation site. The possibility of compensatory effects on replication by the flanking sequences was excluded by an additional experiment, in which mutants containing deletions of 281 nucleotides upstream of nt 2581 or 823 nucleotides downstream of nt 2619 exhibited high level accumulations of RNA similar to that of TNV-A^C^ (Fig. S2A and S2B). Also, western-blot results with antibodies raised against to the P23 replication protein showed that *in vitro* translation of the polymerase in a wheat germ extract is not affected by deletions in the internal sequence or its upstream region, although deletions within the 2609 to 2617 nt region reduced or eliminated viral RNA synthesis (Fig. 4, 5 and 6). Interestingly, levels of the P23 protein translated from pTCPΔ831 RNA containing a deletion of the entire CP region (nt 2612–3442) were significantly higher than those from wtTNV-A^C^ RNAs or other mutants with deletions either upstream or downstream of the regulatory RNA sequence (nt 2609–2617). The latter results possibly reflect negative regulation of polymerase translation by sequences within the 2609 to 2617 nt region (Fig. S2C).

### Translational enhancement by the RNA element

**Figure 7:**
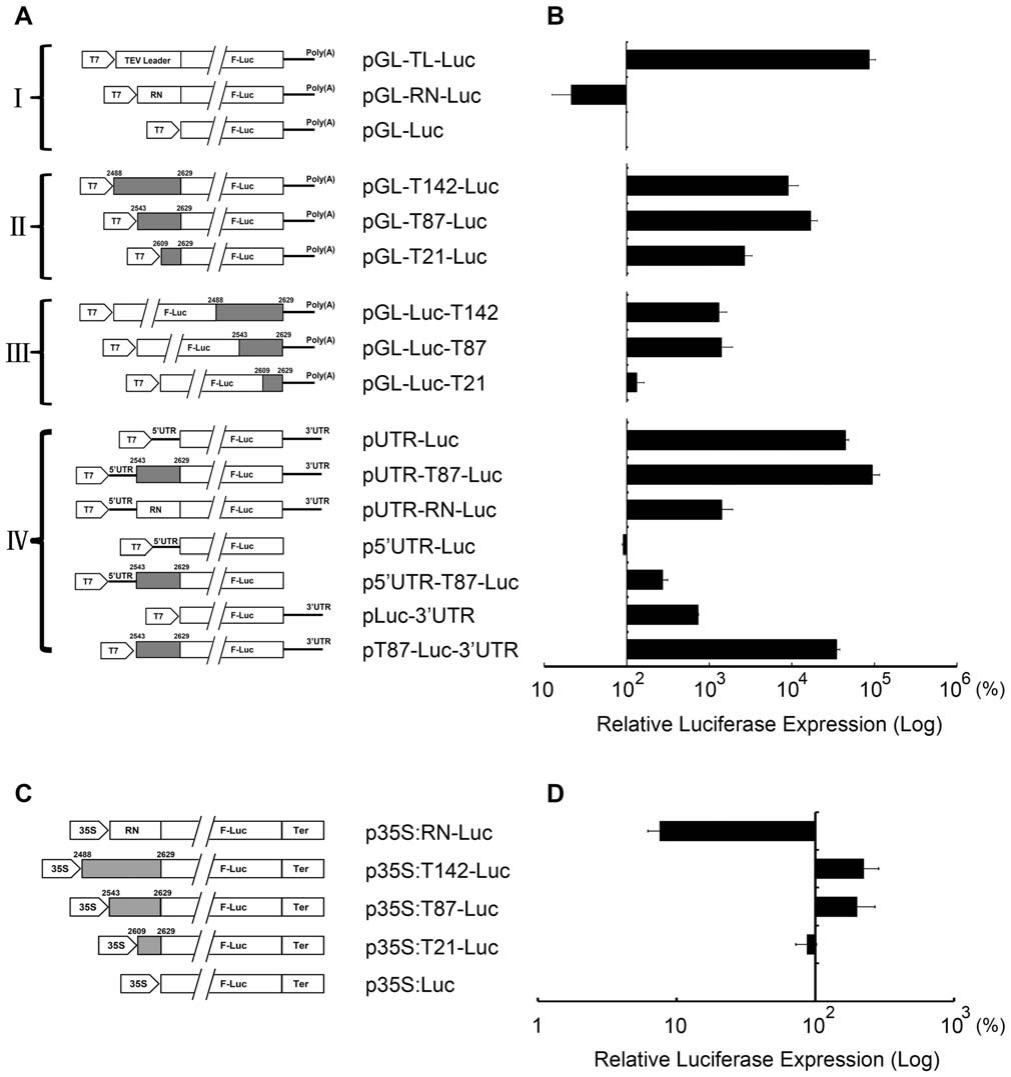
Luciferase assay of the translation enhancement by the investigated elements in protoplasts. (A) Schematic illustrations of firefly luciferase (F-Luc) expression vectors for transcription of chimaeric RNAs and protoplast transfections are shown above. The full-length F-Luc gene used for expression is represented by white boxes interrupted by two lines to reduce the length of the illustrations. The gray boxes represent TNV-A^C^ fragments of different lengths that are indicated by their nucleotide positions relative to TNV RNA. The pentagons represent the T7 promoters and the solid lines indicate poly (A) tails of the mRNA transcripts. The designations upstream of the Luc sequences are: TEV Leader  =  TEV leader sequences involved in translational enhancement, RN  = 81 nonviral nucleotides derived from the plasmid pGL3-Basic, 5′ UTR  =  TNV-A^C^ 5′ UTR sequence, 3′ UTR  =  TNV-A^C^ 3′ UTR sequence. Roman numerals I to IV represent the construct categories for the uncapped mRNA transcripts used for F-Luc expression. (B) Relative F-Luc expression in BY-2 protoplasts was assayed as described in the text, and the results represent an average of three independent experiments, each of which was carried out in triplicate. Expression of the *Renilla* luciferase (R-Luc) from the plasmid pRL-TK served as an internal control throughout the experiments. (C) DNA plasmids for transient expression of firefly luciferase (F-Luc) are illustrated, and the symbols have the same meanings as those in (A), except that the pentagon represents the 35S promoter, and the *Cauliflower mosaic virus* terminator sequences are indicated. (D) Relative F-Luc expression detected as in (B).

As shown in Fig. 3, the internal sequence upstream of the TNV-A^C^ CP gene contains an RNA element necessary for transient GUS expression. To determine whether this element plays a role in cap-independent translation, luciferase reporters were expressed in BY-2 cells transfected with uncapped chimaeric RNAs synthesized *in vitro* or with plant expression vectors containing the TNV-A^C^ fragments driven by the 35S promoter of *Cauliflower mosaic virus* (CaMV). First, the viral fragments were fused with a firefly luciferase (F-Luc) gene in the Dual-luciferase assay system to quantitatively evaluate translational efficiency. Considering the potential interference from the upstream sequence as shown in Figure 3 and Figure 5C, three segments of 21 (nt 2609–2629), 87 (nt 2543–2629) and 142 (nt 2488–2629), corresponding to the lengths of those used in GUS expression, were integrated into the 5′ or 3′ ends of the F-Luc gene (Fig. 7A). These constructs and the internal control *Renilla* luciferase (R-Luc) from pRL-TK were co-transfected into BY-2 protoplasts. The relative luciferase activities of each construct were analysed at 16 hpt, and normalised to values for the pGL-Luc control vector, which was defined as 100%. To preclude an effect from the sequence insertion, pGL-RN-Luc, a construct that contained 81 nucleotides (RN) from the plasmid pGL3-Basic was used as a negative control. As shown in Fig. 7B–II, the pGL-T87-Luc construct stimulated F-Luc translation to the highest level (∼170-fold more than the control), while pGL-T142-Luc and pGL-T21-Luc also stimulated translation (∼90-fold and 27-fold, respectively), whereas pGL-RN-Luc resulted in about a 20% reduction compared to the control. Consistent with these results, a similar but less dramatic pattern of F-Luc expression was observed when the fragments were inserted at the 3′ end of the F-Luc genes; pGL-Luc-T87 and pGL-Luc-T142 increased translation by >13-fold compared to the control, respectively, whereas pGL-Luc-T21 had an effect comparable to the control (Fig. 7B–III). Compared with the F-Luc activity in Fig. 7B–II, the lower translation efficiencies suggest that the RNA element exerts a positional effect on expression when located at 3′ end of F-Luc. Overall, these results are consistent with those from the GUS expression experiments (Fig. 3), and suggest that an RNA element between nt 2543 and the 17-nt region promotes translation as an independent enhancer when located at either the 5′ or 3′ ends of the reporter gene. By integrating these results (Fig. 4–6), we demonstrate that the RNA element is primarily composed of a 9-nt key motif (nt 2609–2617) that is essential for regulation of replication, and that the sequence extending upstream to nt 2543 is necessary for efficient translation.

Because an interaction between the 5′ UTR and the BTE in the 3′ UTR of TNV has been reported to facilitate efficient cap-independent translation [15], [17], the optimally functional 87 nt fragment (nt 2543–2629) was reconstructed in pUTR-T87-Luc, p5′UTR-T87-Luc and pT87-Luc-3′UTR. Both 5′ and 3′ TNV-A^C^ UTR constructs were generated (Fig. 7A–IV), to confirm whether the putative RNA element could enhance 5′-3′ RNA-RNA interaction needed to stimulate translational efficiency. After electroporation of *in vitro* synthesised transcripts, relative luciferase activities in cells transfected by pUTR-T87-Luc and pT87-Luc-3′UTR were elevated significantly (Fig. 7B–IV). We compared the pGL-T87-Luc, pUTR-Luc and p5′UTR-T87-Luc constructs that contain the 87 nt fragment or the 5′ and 3′ UTRs alone, or only the 5′ UTR fused with the 87 nt fragment, respectively (Fig. 7B–II and 7B–IV). This experiment demonstrated that luciferase translation was stimulated synergistically by involvement of the TNV-A^C^ UTRs, possibly resulting mainly from interactions between the 87 nt region and the BTE in the 3′ UTR.

To further determine whether the element plays a role in transcriptional enhancement, three TNV-A^C^ fragments containing 21, 87 or 142 nts were fused to the 5′ end of F-Luc in the 35S promoter driven plant expression pRTL2 vector, and two constructs (p35S:RN-Luc and p35S:Luc) were created as controls (Fig. 7C). Upon transient expression of the chimaeric DNAs in BY-2 cells, the relative luciferase activity in cells transfected with p35S:T142-Luc or p35S:T87-Luc was about two-fold higher than the p35S:Luc positive control, but was slightly reduced in cells transfected with p35S:T21-Luc (Fig. 7D). In contrast to the significant reduction in efficiency caused by the insertion of the RN sequence in p35S:RN-Luc, the results suggest that extended viral sequences upstream of the CP gene may contain a transcription factor binding site necessary for optimal RNA synthesis. However, a truncated version consisting 21 nts (nt 2609–2629) was not functional (Fig. 7C and 7D), and this confirms a length-dependent requirement for enhancement by the element (as shown in Fig. 7A and 7B).

In summary, we have identified an RNA regulatory element in the internal region of TNV-A^C^ genome that ranges from approximately nt 2543 to nt 2617. This fragment contains a 9-nt key domain (nt 2609–2617) that is essential for virus replication and high efficiency expression of reporter genes in plant leaves or in tobacco cells. The element is associated with viral UTRs, mainly with the 3′ UTR and these associations are required for synergistic enhancement.

## Discussion

The CP's of plant viruses frequently have multiple functions during infection [26] and are critical determinants involved in viral pathogenicity. In the *Tombusviridae*, reduced levels of *Turnip crinkle virus* (TCV) CP are responsible for sat-RNA mediated symptom attenuation [27], the TCV CP also acts as a suppressor early in initiation of post-transcriptional gene silencing [28], [29], and two amino acids at the N terminus of the TCV CP are involved in eliciting a hypersensitive response (HR) in Di-17 *Arabidopsis* [30]. In addition, an untranslated RNA sequence in the *Cymbidium ringspot tombusvirus* (CymRSV) CP gene triggers an HR-like resistance response in *Datura stramonium* [31]. Similar phenomena have been described previously by our group with TNV-A^C^ [22] and *Beet black scorch virus* (BBSV) [32]. For example, local lesion symptoms in *C. amaranticolor* were attenuated or delayed when the CP initiation codons were mutated or intact genes were deleted. In contrast, a reverse mutation of the CP start codon from AG^2613^G back to AUG in the construct pTCPM-I lead to a restoration of severe lesion phenotype similar to that of wt TNV-A^C^ [22]. Serial deletion experiments eliminated the possibility that the TNV-A^C^ CP functions as a silencing suppressor or an HR elicitor. Comparative analysis revealed that a 9-nt key oligonucleotide adjacent to the CP initiation site (nt 2609–2617) in TNV-A^C^ also occupies the same position in other TNV-A isolates [33], [34]. Similar sequence motifs with one or two nucleotide alterations are present in the CP ORFs of some *Tombusviridae* family members, including BBSV [35], [36], TBSV [37], [38], *Carnation Italian ringspot virus* [39], CymRSV [40] and TCV [41]. We suggest that viruses in the family *Tombusviridae* contain a highly-conserved sequence in the vicinity of the CP start codon that is remarkably resistant to spontaneous mutation during evolution. As shown in Figure 5B, expression of sgRNA2 was significantly reduced or abolished in infections with the Δ2312–2373, Δ2374–2429, and Δ2431–2491 mutants, suggesting that in addition to the start site at G^2460^ reported previously [22], the adjacent sequence is involved in initiation of TNV-A^C^ sgRNA2 transcription. Moreover, although alignments of the homologous RNA regions among the necroviruses and tombusviruses showed no significant conservation throughout the 87-nt region, the nucleotides ^2593^UUUC^2596^ in the 87-nt region that exist in some other necroviruses could base-pair with the ^3512^GAAA^3515^ sequences of the BTE stem-loop I motif (^3503^GGAUCCUGAGAAACAGG^3519^) and ^3623^GAAA^3626^ of the 3′ end stem-loop II motif in the 3′UTR. This possible terminal bulge base-paring may be important for formation of the conserved 3′ UTR secondary structure predicted by Na [42] and Shen [16]. Also, our recent result revealed that the guanine composing the ^3512^GAAA^3515^ loop is responsible for the high level CP accumulation and the severer symptoms of BBSV, TNV-A and TNV-D by Xu [43]. In addition, a short nucleotide stretch (^2612^AUGGCA^2617^) is highly conserved amongst necroviruses and tombusviruses. This sequence is partially complementary to the ^3466^UGCC^3469^ or ^3609^GCCA^3612^ sequences in the 3′UTRs, but appear not to be involved in the 5′ and 3′ long-distance interactions described by Shen [15] and Meulewaeter [17].

Various regulatory elements mostly located at the termini of viral genomes are employed by RNA viruses to mediate appropriate timing of gene expression and levels of accumulation. For instance, the internal ribosome entry sites (IRES) in the 5′ UTR of *Tobacco etch virus* (TEV) [44]–[46], *Potato virus Y* (PVY) [47], [48], *Turnip mosaic potyvirus* (TuMV) [49], and *Blackcurrant reversion virus* (BRV) RNA2 [50] act to recruit ribosomes to modestly stimulate translation, whereas structured sequences in the 5′ leader of *Tobacco mosaic virus* (TMV) [51] and TEV [45] promote cap-independent translation. In addition, many plant viruses harbor translational enhancers in their 3′ UTRs that function to regulate translational efficiency through base-pairing interactions with 5′ UTRs, such as the BTE in necroviruses [15]–[17] and the cap-independent translation element (CITE) in tombusviruses [4], [52], [53]. It has been shown that cap-independent translation of TNV-A sgRNA2 requires a synergistic interaction between the 3′ BTE and the 5′ leader [17] located 152 nucleotides upstream of the CP gene [21], and these results reveal an involvement of the sgRNA leader in translational promotion. In our study, integrating the results in Figure 5C, 7 and S2 reveals a sequence ranging from approximately nt 2583 to nt 2617 in the centre of TNV-A^C^ genome, which is composed of the 9 nt key region. An additional sequence upstream of the sgRNA2 leader is also indispensable for viral RNA replication and functions in translational enhancement. Intriguingly, when present in reporter vectors lacking a TNV-A^C^ trailer sequence, the element can also increase the efficiency of protein expression significantly. These results demonstrate that the sequence motif can function independently of a viral context to facilitate translation of heterologous mRNAs.

In addition, the sequence ^2602^CAAAAUAGAC^2611^ present in the TNV-A^C^ enhancer element shares very high similarity with the upstream region ^28^CAAAACAAAC^37^ flanking pseudoknot 1 (PK1) in the TEV 5′-leader, which is necessary for promotion of cap-independent translation [45]. Hence, we presume that the oligonucleotide motif in the TNV-A^C^ genome may be involved in translational enhancement by generating functional structure with other viral sequences.

In addition to the 3′-BTE, we have identified a new element for cap-independent translation in the centre of the TNV-A^C^ gRNA that may provide an alternative mechanism to stimulate protein translation. Distinct functional features of this element still remain to be elucidated and will be a subject for future investigations in our lab.

## Materials and Methods

### Mutant cDNA construction

A full-length infectious cDNA of TNV-A^C^ (pMTC27) [19] was used as a template for construction of deletion mutants (Fig. 1A, 5A and 6A) by inverse Polymerase Chain Reaction (PCR) amplification [54]. PrimeSTAR HS DNA Polymerase (TaKaRa) was used according to the manufacturer's instructions, except that the central portion of the CP gene was removed by digestion with *Aor*51HI and *Eco*RV to construct the mutant pTCPΔ488 (Fig. 1A). All primers used for mutant constructions are listed in Table S1. The PCR products were digested with *Dpn*I to remove template DNA from the mixtures [55], followed by gel-purification, phosphorylation and self-ligation to produce desired mutants. The same strategy was used for dual deletions of mutant pTCPΔ814 (Fig. 4A) and pTCPΔ823 (Fig. S2A) templates.

To generate GUS gene-fused TNV-A^C^ recombinants for plant inoculations, a 1.8 Kb fragment of the GUS open reading frame (ORF) was amplified by PCR from pBGUS [32], using the primers listed in Table S1. Amplified fragments were used as intermediate templates for overlap extension PCR [54], and digested with *Dra*III and *Eco*RI before cloning into pMTC27 to create pTGUS-I (Fig. 3A). The same strategy was employed to construct pTGUS-II, pTGUS-I 17, pTGUS-I 81 and pTGUS-I 294 from the pTGUS-I template (Fig. 3A).

To test the regulation of translational efficiency by the internal viral sequences, a firefly luciferase (F-Luc) reporter was fused upstream or downstream of TNV-A^C^ fragments of various lengths, which were transcribed *in vitro* for tobacco cell inoculation. For these derivatives, the TNV-A^C^ mutant pTCPΔ814 plasmid (Fig. 1A) was used as the template for PCR amplification of the internal fragments, and the F-Luc gene was derived from the plasmid pGL3-Promoter (Promega). Four categories of the mutant constructs driven by a T7 promoter were made by inverse PCRs, cDNA ligations and restriction digestions through various intermediate structures (Fig. 7A). Six of the constructs contained internal viral sequences of 142 nts (nt 2488–2629), 87 nts (nt 2543–2629) or 21 nts (nt 2609–2629), either upstream (pGL-T142-Luc, pGL-T87-Luc and pGL-T21-Luc, in category II) or downstream (pGL-Luc-T142, pGL-Luc-T87 and pGL-Luc-T21, in category III) of the F-Luc ORF (Fig. 7A–II and 7A–III). In category I, the pGL-Luc construct, which lacked a viral sequence insertion, and pGL-RN-Luc, which contained 81 nucleotides (designated RN) from the plasmid pGL3-Basic (Promega), were used as positive or negative controls, respectively (Fig. 7A–I). In a separate reaction based on the construct pGL-Luc, the *Tobacco etch virus* (TEV) leader sequence from plasmid pRTL2 [44] was ligated upstream of the F-Luc gene to generate the pGL-TL-Luc construct for comparisons with the TNV-A^C^ sequences for translational enhancement activities (Fig. 7A–I). To test possible synergistic functions of the internal sequence, the 5′ and/or 3′ UTR regions of the TNV-A^C^ genome were inserted into the construct pGL-T87-Luc in sequential positions, to provide category IV constructs, pUTR-T87-Luc, p5′UTR-T87-Luc and pT87-Luc-3′UTR, while the pUTR-RN-Luc construct was generated for use as a negative control (Fig. 7A–IV).

For transient expression in tobacco cells, the 35S promoter-driven constructs were created by transferring expression fragments from pGL-T142-Luc, pGL-T87-Luc or pGL-T21-Luc into the plant expression vector pRTL2 [44] to produce p35S:T142-Luc, p35S:T87-Luc and p35S:T21-Luc derivatives (Fig. 7C). The same strategy was used to generate the controls, p35S:Luc and p35S:RN-Luc (Fig. 7C). All of the derivatives were verified by cDNA sequencing, and a *Renilla* luciferase (R-LUC) gene from pRL-TK (Promega) driven by T7 or 35S promoter was used as a control throughout the experiments.

### Growth of plants and tobacco cells for inoculations

*C. amaranticolor* plants used in this experiment were grown in a growth chamber at 24°C with a 14-hour-light/10-hour-dark cycle. Tobacco BY-2 (*N. tabacum* L. var Bright Yellow 2) suspension cells [56] were incubated in growth shaker at 130 rpm without light at 24°C, and subcultured weekly.

### *In vitro* synthesis of RNA and inoculations

Viral RNAs were synthesised *in vitro* with T7 RNA polymerase (Promega) and purified with HiBind® spin cartridges (OMEGA) according to the manufacturer's instructions. RNA concentrations were determined by spectrophotometry (NanoDrop Technologies, Inc.), and RNA integrity was assessed by 1% agarose gel electrophoresis. One to two μg of *in vitro* synthesised RNAs were mixed with an equal volume of inoculation buffer (50 mM glycine, 30 mM K_2_HPO_4_, 1% bentonite, 1% celite, pH 9.2) for mechanical inoculation of *C. amaranticolor* leaves. Transfection of protoplasts was carried out as described previously [56], [57]. Briefly, protoplasts (2×10^6^ cells/ml) isolated from BY-2 suspension cells were electroporated with 10 μg of the viral RNA transcripts and incubated in the dark at 24°C for 18 hours. To eliminate background interference, a mock treatment was performed by mixing the protoplasts with RNA transcripts without electroporation.

### RNA and protein analysis

Total RNA was isolated from leaves of *C. amaranticolor* at 4 days post-inoculation (dpi) as previously described [22], [58], [59], or from transfected tobacco protoplasts at 18 hours post-transfection (hpt) by extraction with TRIZOL Reagent as recommended by the manufacturer (Invitrogen). The viral RNAs were subjected to Northern blot analysis with a cDNA probe specific to the 3′ UTR of TNV-A^C^, prepared as in previous reports [22], [54]. Viral cDNAs were amplified by RT-PCR from the total RNA extracted from infected plants or protoplasts to identify reverse mutation that might have occurred during replication. Total protein extracted from transfected protoplasts was subjected to immunoblot analysis with a specific antibody (P30) raised against the TNV-A^C^ CP [22].

### Dual-Luciferase reporter assay

BY-2 protoplasts (1.0×10^6^ cells/ml) were co-electroporated with 15 μg of uncapped RNA transcripts, or 20 μg of DNA containing a F-Luc gene or 1.5–2 μg of the R-Luc internal control and then were incubated at 24°C for 16 h. Cells were harvested by centrifugation at 150 g for 3 min at room temperature, washed with phosphate buffered saline (PBS), and centrifuged at 10,000 g for 3 min at 4°C. The pellet was lysed in 250 μl of passive lysis buffer (Promega) before being subjected to one or two freeze-thaw cycles to completely disrupt the cells. Aliquots (20 μl) of cell lysate were measured for firefly and *Renilla* luciferases activities with a GloMax® 20/20 Luminometer (Promega), in which light readings of extracts from mock-inoculated protoplasts were used as background controls. The relative luciferase activities of the constructs were normalised with *Renilla* luciferase standard and expressed as a percentage relative to the pGL-Luc or p35S:Luc controls, which were set as 100%.

## Supporting Information

Figure S1 **Serial deletions within the 136-nt region at the 5′ end of the TNV-A^C^ coat protein gene and plant inoculations.** (A) The organisation of wtTNV-A^C^ (pMTC27) ORFs are illustrated by gray boxes. The region at the 5′ end of the CP ORF is indicated by solid lines to illustrate the sequence used for the deletions, and the deleted sequences are indicated by dashed lines with numbers identifying the deleted sequences appearing above the dotted regions. Designations of the eight deletion mutants and the nucleotide deletions are shown on the left side of the figure. (B) The inoculated *C. amaranticolor* leaf phenotype was photographed at 4 dpi, and the mutant viral RNAs used for inoculations are shown above each photo. (C) Viral RNA accumulation in *C. amaranticolor* plants inoculated by the respective mutants was assessed by Northern blots with the same probe used in Fig. 1. TNV-A^C^ RNA species are indicated on the left side of the gel photographs and plant rRNAs are shown as loading controls in the bottom panel.
(TIF)

Figure S2 **Viral RNA accumulations in inoculated tobacco BY-2 protoplasts and** ***in vitro*** **translation assay of the P23 replicase protein with mutant TNV-A^C^ RNAs containing deletions of the internal region.** (A) Schematic representation of pTCPΔ823 (see Fig. 4A) and four deletion mutants derived from pTCPΔ823. The dotted angular region and the four mutant designations to the left side of the figure identify sequences deleted from the mutagenized pTCPΔ823 region (nt 2620–3442), where the deleted nucleotides range from nt 2301 to 2608. (B) Viral RNA accumulation in tobacco BY-2 protoplasts assessed by Northern blots with the same probe used in Fig. 1. TNV-A^C^ RNA species are indicated on the left side of the gel photographs and plant rRNAs used as loading controls are shown in the bottom panel. (C) Western blot analysis of P23 replicase proteins synthesized during *in vitro* translation with wheat germ extract (Promega). Viral constructs used for *in vitro* mRNA synthesis are indicated above each lane in panels B and C (see Fig. 1A and 5A). A specific antibody (P23) raised against TNV-A^C^ replication protein was used for protein analysis. The mock lane consists of a translation reaction with no RNA added. The position of the expected P23 product is indicated to the left and ribosomal protein isolated from the extract provides a loading control. Products were generated by translating 15 μg of uncapped full-length viral genome transcripts in wheat germ extract for 1.5 hr at 25°C.
(TIF)

Table S1 **Primers used in this study.**
(PDF)
