# Supplementary figures and images for: Identification of an Internal RNA Element Essential for Replication and Translational Enhancement of Tobacco Necrosis Virus A C
Source: PLoS One. 2013 Feb 27;8(2):e57938. doi: 10.1371/journal.pone.0057938 (PMC3583896; doi:10.1371/journal.pone.0057938)

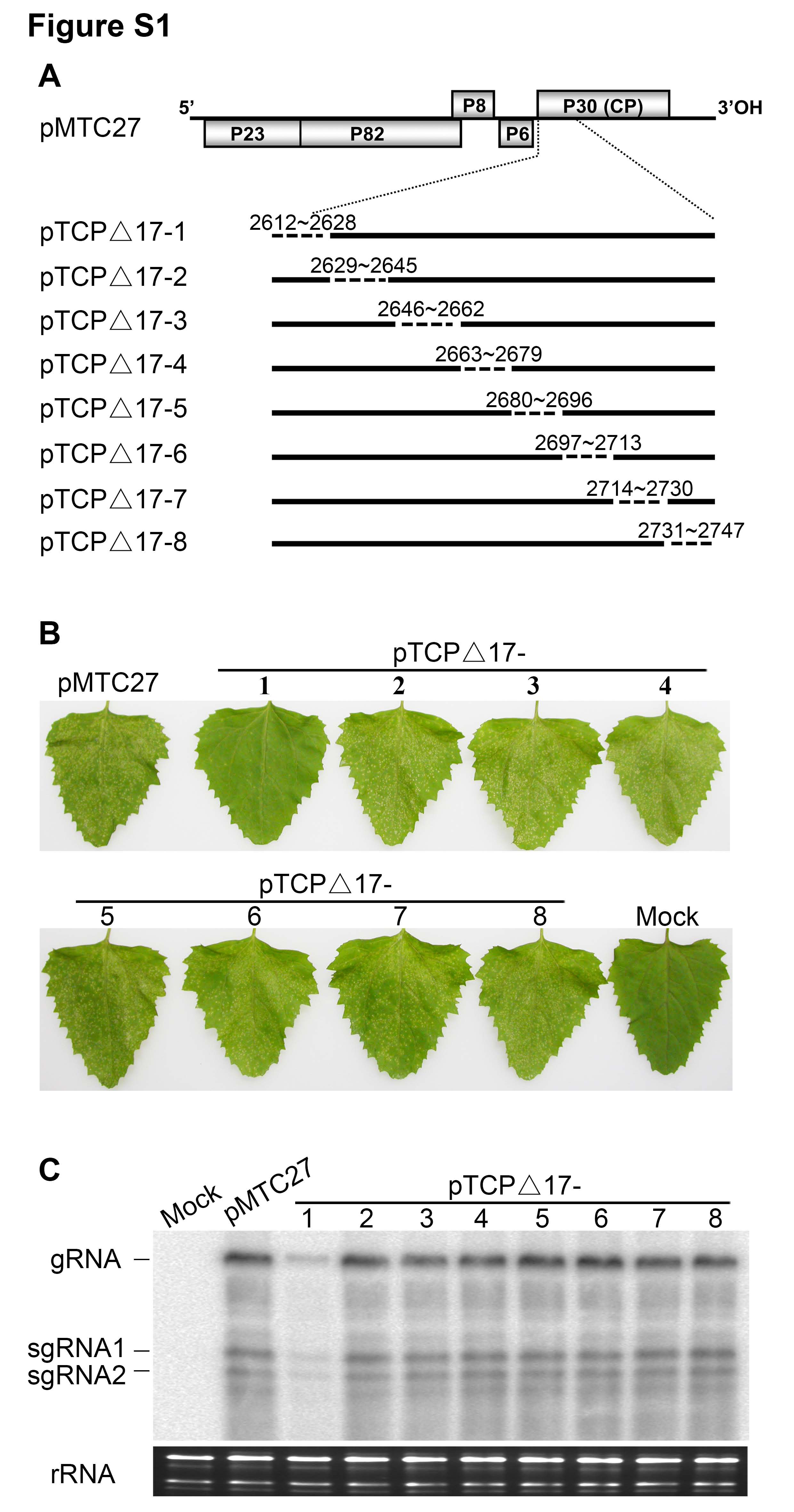

Supplement: Figure S1 — Serial deletions within the 136-nt region at the 5′ end of the TNV-AC coat protein gene and plant inoculations. (A) The organisation of wtTNV-AC (pMTC27) ORFs are illustrated by gray boxes. The region at the 5′ end of the CP ORF is indicated by solid lines to illustrate the sequence used for the deletions, and the deleted sequences are indicated by dashed lines with numbers identifying the deleted sequences appearing above the dotted regions. Designations of the eight deletion mutants and the nucleotide deletions are shown on the left side of the figure. (B) The inoculated C. amaranticolor leaf phenotype was photographed at 4 dpi, and the mutant viral RNAs used for inoculations are shown above each photo. (C) Viral RNA accumulation in C. amaranticolor plants inoculated by the respective mutants was assessed by Northern blots with the same probe used in Fig. 1. TNV-AC RNA species are indicated on the left side of the gel photographs and plant rRNAs are shown as loading controls in the bottom panel. (TIF) [file pone.0057938.s001.tif]

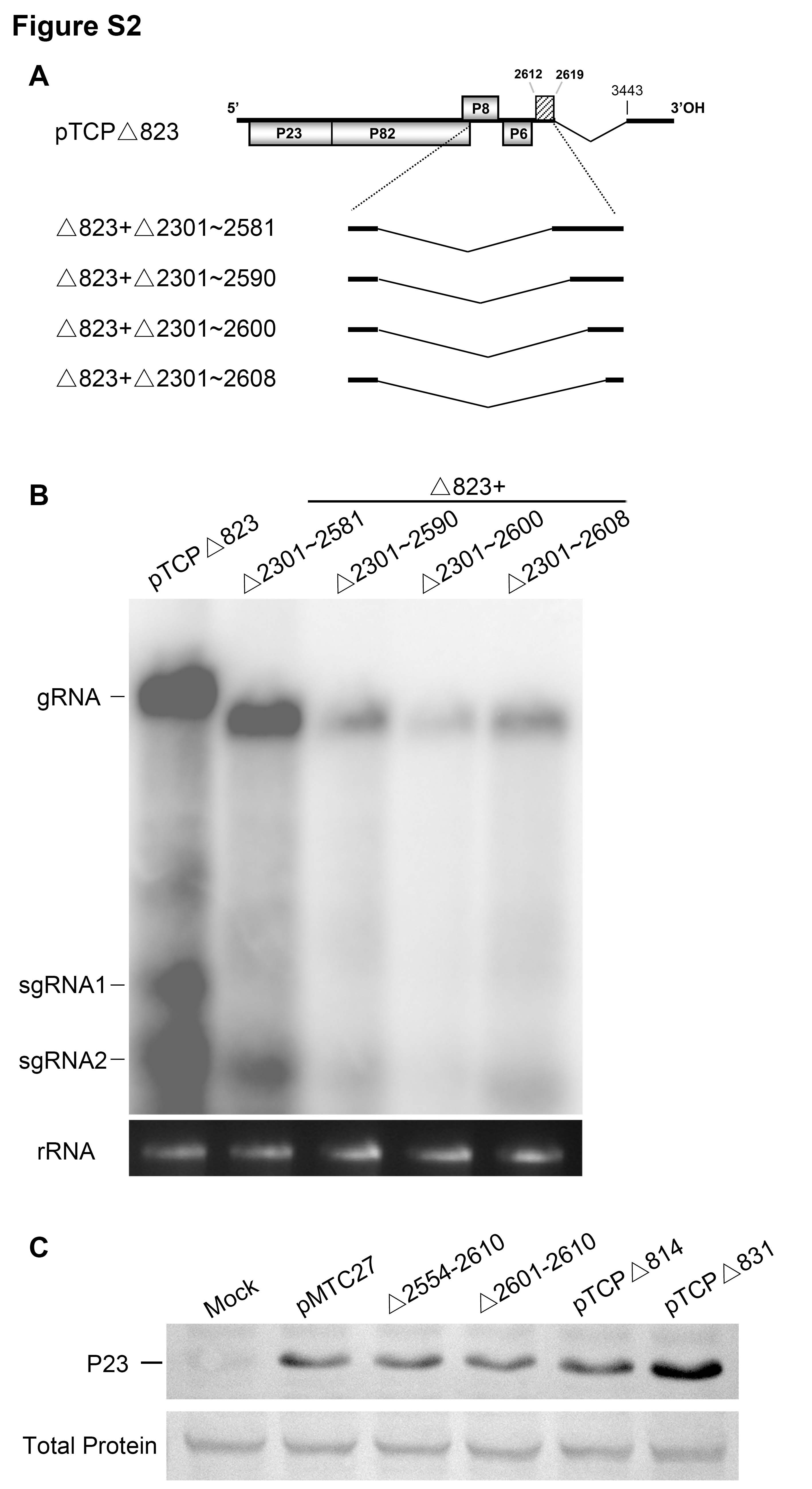

Supplement: Figure S2 — Viral RNA accumulations in inoculated tobacco BY-2 protoplasts and in vitro translation assay of the P23 replicase protein with mutant TNV-AC RNAs containing deletions of the internal region. (A) Schematic representation of pTCPΔ823 (see Fig. 4A) and four deletion mutants derived from pTCPΔ823. The dotted angular region and the four mutant designations to the left side of the figure identify sequences deleted from the mutagenized pTCPΔ823 region (nt 2620–3442), where the deleted nucleotides range from nt 2301 to 2608. (B) Viral RNA accumulation in tobacco BY-2 protoplasts assessed by Northern blots with the same probe used in Fig. 1. TNV-AC RNA species are indicated on the left side of the gel photographs and plant rRNAs used as loading controls are shown in the bottom panel. (C) Western blot analysis of P23 replicase proteins synthesized during in vitro translation with wheat germ extract (Promega). Viral constructs used for in vitro mRNA synthesis are indicated above each lane in panels B and C (see Fig. 1A and 5A). A specific antibody (P23) raised against TNV-AC replication protein was used for protein analysis. The mock lane consists of a translation reaction with no RNA added. The position of the expected P23 product is indicated to the left and ribosomal protein isolated from the extract provides a loading control. Products were generated by translating 15 μg of uncapped full-length viral genome transcripts in wheat germ extract for 1.5 hr at 25°C. (TIF) [file pone.0057938.s002.tif]
