# Supplementary material for: Identification of an Internal RNA Element Essential for Replication and Translational Enhancement of Tobacco Necrosis Virus A C
Source: PLoS One. 2013 Feb 27;8(2):e57938. doi: 10.1371/journal.pone.0057938 (PMC3583896; doi:10.1371/journal.pone.0057938)
Supplement: Table S1 — Primers used in this study. (PDF) [file pone.0057938.s003.pdf]

Supplemental Table 1. Primers used in this study

Primer	Primer sequence (5′–3′)	Position and description [a]	Note of usage
TN-18	GGGTGGGGCAAAAGCCCCTCAAT	Reverse complementary to TNV-A^C nt 3660-3682	For RT-PCR assay with TN-421F
TN-64	CGGAATTCCGCCCGGGGTGGGGCAAAAG CCC	Reverse complementary to TNV-A^C nt 3665-3682, *Sma*I and *Eco*RI and their terminal nucleotides are underlined	Construction of the GUS-fused recombinant viruses
TN-102F	GCTACATCAACGAGCATGGGC	Corresponding to TNV-A^C nt 2748-2768	Construction of pTCPΔ136 based on pMTC27 with TN-103R
TN-103R	GTCTATTTTGCGATCGAAATG	Reverse complementary to TNV-A^C nt 2591-2611	
TN-104F	TTCTTGTACTGTAACTCGACC	Corresponding to TNV-A^C nt 3443-3463	Construction of pTCPΔ207 based on pMTC27 with TN-105R
TN-105R	ATCCAACTGGATAGCAATGGC	Reverse complementary to TNV-A^C nt 3215-3235	
TN-106F	GCTACATCAACGAGCATGGG	Corresponding to TNV-A^C nt 2748-2766	Construction of pTCPΔ17-8 based on pMTC27 with TN-107R
TN-107R	ATTTTCTTCATGCGGCCTCG	Reverse complementary to TNV-A^C nt 2711-2730	
TN-108F	GAGGCAGCCAGTTCAGCGCT	Corresponding to TNV-A^C nt 2731-2750	Construction of pTCPΔ17-7 based on pMTC27 with TN-109R
TN-109R	TCGTGCCAGCCTGCGTGCATC	Reverse complementary to TNV-A^C nt 2693-2713	
TN-110F	GGCCGCATGAAGAAAATGAG	Corresponding to TNV-A^C nt 2714-2733	Construction of pTCPΔ17-6 based on pMTC27 with TN-111R
TN-111R	CATCACGCTGGTCAATTTCC	Reverse complementary to TNV-A^C nt 2677-2696	
TN-112F	CACGCAGGCTGGCACGAGGC	Corresponding to TNV-A^C nt 2697-2716	Construction of pTCPΔ17-5 based on pMTC27 with TN-113R
TN-113R	GTCCACCTGTTGCTCTGGAG	Reverse complementary to TNV-A^C nt 2661-2679	
TN-114F	AATTGACCAGCGTGATGCAC	Corresponding to TNV-A^C nt 2680-2699	Construction of pTCPΔ17-4 based on pMTC27 with TN-115R
TN-115R	AGTACGCAGTACAATGTAC	Reverse complementary to TNV-A^C nt 2643-2662	
TN-116F	CCAGAGCAACAGGTGGAAAT	Corresponding to TNV-A^C nt 2663-2682	Construction of pTCPΔ17-3 based on pMTC27 with TN-117R
TN-117R	ACTGACCATTGTTGTTGTTC	Reverse complementary to TNV-A^C nt 2626-2645	
TN-118F	ACATTGTACTGCGTACTCCAG	Corresponding to TNV-A^C nt 2646-2666	Construction of pTCPΔ17-2 based on pMTC27 with TN-119R
TN-119R	TTCTTCTTTCCTGCCATGTC	Reverse complementary to TNV-A^C nt 2609-2628	
TN-120F	CAACAACAATGGTCAGTAC	Corresponding to TNV-A^C nt 2629-2647	Construction of pTCPΔ17-1 based on pMTC27 with TN-121R

TN-121R	GTCTATTTTGCGATCGAAATG	Reverse complementary to TNV-A^{C} nt 2591-2611	
TN-132	GGGCTAGGAGTAGTGAGGGTA AG	Corresponding to TNV-A^{C} nt 2267-2289	Construction of the GUS-fused recombinant viruses
TN-134R	CAAGAATTCTTCTTTCCTGCCATGTC	Reverse complementary to TNV-A^{C} nt 2609-2628	Construction of pTCPΔ814 based on pMTC27 with TN-135F
TN-135F	TTCTTGTACTGTAACTCGACC	Corresponding to TNV-A^{C} nt 3443-3463	
TN-290F	TTCTTGTACTGTAACTCGACCAATGCC	Corresponding to TNV-A^{C} nt 3443-3469	Preparation of the template for the DNA probe of Nothern blot with TN18
TN-421F	CTAGATTCATGAATAGAGTGGCCCG	Corresponding to TNV-A^{C} nt 2033-2059	For RT-PCR assay with TN-18
TN-426R	TCAATCCCGAACCATTGTGTTTCTTAC	Reverse complementary to TNV-A^{C} nt 2285-2311	Construction of Δ2312-2373 based on pMTC27 with TN-427F
TN-427F	GGAACAATGACCAACATAGCCGAAGAG	Corresponding to TNV-A^{C} nt 2374-2400	
TN-428R	GTTGCCAGTACCTTGCTGTGATTTCTG	Reverse complementary to TNV-A^{C} nt 2347-2373	Construction of Δ2374-2429 based on pMTC27 with TN-429F
TN-429F	CTTTTAGTTATGGCGTATTGTCGCTGT	Corresponding to TNV-A^{C} nt 2430-2456	
TN-430R	GTTAAAGTTGTATGTCACGGTAATGGT	Reverse complementary to TNV-A^{C} nt 2404-2430	Construction of Δ2431-2491 based on pMTC27 with TN-431F
TN-431F	CTTTGCAATTCTCATCCTCATATTAGC	Corresponding to TNV-A^{C} nt 2492-2518	
TN-432R	GTAGGGGAATAATGTAATACCTGGTGA	Reverse complementary to TNV-A^{C} nt 2466-2492	Construction of Δ2493-2552 based on pMTC27 with TN-433F
TN-433F	CATCATTCTCCTAGTACTTACGAGTAT	Corresponding to TNV-A^{C} nt 2553-2579	
TN-434R	GATATTGTTGGTTTGGAGTGCCAACAA	Reverse complementary to TNV-A^{C} nt 2527-2553	Construction of Δ2554-2610 based on pMTC27 with TN-435F
TN-435F	CATGGCAGGAAAGAAGAACAACAACAA	Corresponding to TNV-A^{C} nt 2611-2637	
TN-448R	ATATTGTTGGTTTGGAGTGCCAACAAC	Reverse complementary to TNV-A^{C} nt 2526-2552	Construction of Δ2553-2562 based on pMTC27 with TN-449F
TN-449F	CTAGTACTTACGAGTATAAGACTCAAC	Corresponding to TNV-A^{C} nt 2563-2589	
TN-450R	GAGAATGATGATATTGTTGGTTTGGAG	Reverse complementary to TNV-A^{C} nt 2536-2562	Construction of Δ2563-2572 based on pMTC27 with TN-451F
TN-451F	CGAGTATAAGACTCAACACATTTCGAT	Corresponding to TNV-A^{C} nt 2573-2599	
TN-452R	TAAGTACTAGGAGAATGATGATATTGT	Reverse complementary to TNV-A^{C} nt 2546-2572	Construction of Δ2573-2582 based on pMTC27 with TN-453F
TN-453F	ACTCAACACATTTCGATCGCAAAATAG	Corresponding to TNV-A^{C} nt 2583-2609	
TN-454R	CTTATACTCGTAAGTACTAGGAGAATG	Reverse complementary to TNV-A^{C} nt 2556-2582	Construction of Δ2583-2590 based on pMTC27 with TN-455F

TN-455F	CATTTCGATCGCAAAATAGACATGGCA	Corresponding to TNV-A^C nt 2591-2617	
TN-456R	GTTGAGTCTTATACTCGTAAGTACTAG	Reverse complementary to TNV-A^C nt 2563-2589	Construction of Δ2590-2600 based on pMTC27 with TN-457F
TN-457F	GCAAAATAGACATGGCAGGAAAGAAGA	Corresponding to TNV-A^C nt 2601-2627	
TN-458R	GATCGAAATGTGTTGAGTCTTATACTC	Reverse complementary to TNV-A^C nt 2574-2600	Construction of Δ2601-2610 with TN-435F based on pMTC27
TN-464R	TCTTCTTTCCTGCCATGTCTATTTTGC	Reverse complementary to TNV-A^C nt 2601-2627	Construction of pTCPΔ815 with TN-290F based on pMTC27
TN-465R	CTTCTTTCCTGCCATGTCTATTTTGCG	Reverse complementary to TNV-A^C nt 2600-2626	Construction of pTCPΔ816 with TN-290F based on pMTC27
TN-466R	TTCTTTCCTGCCATGTCTATTTTGCGA	Reverse complementary to TNV-A^C nt 2599-2625	Construction of pTCPΔ817 with TN-290F based on pMTC27
TN-467R	TCTTTCCTGCCATGTCTATTTTGCGAT	Reverse complementary to TNV-A^C nt 2598-2624	Construction of pTCPΔ818 with TN-290F based on pMTC27
TN-468R	CTTTCCTGCCATGTCTATTTTGCGATC	Reverse complementary to TNV-A^C nt 2597-2623	Construction of pTCPΔ819 with TN-290F based on pMTC27
TN-469R	TTTCCTGCCATGTCTATTTTGCGATCG	Reverse complementary to TNV-A^C nt 2596-2522	Construction of pTCPΔ820 with TN-290F based on pMTC27
TN-470R	TTCCTGCCATGTCTATTTTGCGATCGA	Reverse complementary to TNV-A^C nt 2595-2621	Construction of pTCPΔ821 with TN-290F based on pMTC27
TN-471F	AAAATAGACATGGCAGGAAAGAAGAAC	Corresponding to TNV-A^C nt 2603-2629	Construction of Δ2601-2602 with TN458R based on pMTC27
TN-472F	AATAGACATGGCAGGAAAGAAGAACAA	Corresponding to TNV-A^C nt 2605-2631	Construction of Δ2603-2604 based on pMTC27 with TN-473R
TN-473R	GCGATCGAAATGTGTTGAGTCTTATAC	Reverse complementary to TNV-A^C nt 2576-2602	
TN-474F	TAGACATGGCAGGAAAGAAGAACAACA	Corresponding to TNV-A^C nt 2607-2633	Construction of Δ2605-2606 based on pMTC27 with TN-475R
TN-475R	TTGCGATCGAAATGTGTTGAGTCTTAT	Reverse complementary to TNV-A^C nt 2578-2604	
TN-476F	GACATGGCAGGAAAGAAGAACAACAAC	Corresponding to TNV-A^C nt 2609-2635	Construction of Δ2607-2608 based on pMTC27 with TN-477R
TN-477R	TTTTGCGATCGAAATGTGTTGAGTCTT	Reverse complementary to TNV-A^C nt 2580-2606	
TN-478R	TATTTTGCGATCGAAATGTGTTGAGTC	Reverse complementary to TNV-A^C nt 2582-2608	Construction of Δ2609-2610 with TN-435F based on pMTC27
TN-479F	ATAGACATGGCAGGAAAGAAGAACAAC	Corresponding to TNV-A^C nt 2606-2632	Construction of Δ2601-2605 with TN-458R based on pMTC27

TN-480R	TTTGCGATCGAAATGTGTTGAGTCTTA	Reverse complementary to TNV-A^{C} nt 2579-2605	Construction of Δ2606-2610 with TN-435F based on pMTC27
TN-499F	GCAAAATAGACATGGCAGGTTCTTGTACTG	Corresponding to TNV-A^{C} nt 2601-2619 and nt 3443-3453	Construction of pTCPΔ823 with TN-508R based on pMTC27
TN-500F	GCAAAATAGACATGGCAGTTCTTGTACTGT	Corresponding to TNV-A^{C} nt 2601-2618 and nt 3443-3454	Construction of pTCPΔ824 with TN-508R based on pMTC27
TN-501F	GCAAAATAGACATGGCATTCTTGTACTGTA	Corresponding to TNV-A^{C} nt 2601-2617 and nt 3443-3455	Construction of pTCPΔ825 with TN-508R based on pMTC27
TN-502F	GCAAAATAGACATGGCTTCTTGTACTGTAA	Corresponding to TNV-A^{C} nt 2601-2616 and nt 3443-3456	Construction of pTCPΔ826 with TN-508R based on pMTC27
TN-503F	GCAAAATAGACATGGTTCTTGTACTGTAAC	Corresponding to TNV-A^{C} nt 2601-2615 and nt 3443-3457	Construction of pTCPΔ827 with TN-508R based on pMTC27
TN-504F	GCAAAATAGACATGTTCTTGTACTGTAACT	Corresponding to TNV-A^{C} nt 2601-2614 and nt 3443-3458	Construction of pTCPΔ828 with TN-508R based on pMTC27
TN-505F	GCAAAATAGACATTTCTTGTACTGTAACTC	Corresponding to TNV-A^{C} nt 2601-2613 and nt 3443-3459	Construction of pTCPΔ829 with TN-508R based on pMTC27
TN-506F	GCAAAATAGACATTCTTGTACTGTAACTCG	Corresponding to TNV-A^{C} nt 2601-2612 and nt 3443-3460	Construction of pTCPΔ830 with TN-508R based on pMTC27
TN-507F	GCAAAATAGACTTCTTGTACTGTAACTCG	Corresponding to TNV-A^{C} nt 2601-2611 and nt 3443-3460	Construction of pTCPΔ831 based on pMTC27 with TN-508R
TN-508R	GATCGAAATGTGTTGAGTCTTATACTCGTA	Reverse complementary to TNV-A^{C} nt 2571-2600	
TN-521R	CTATTTTGCGATCGAAATGTGTTGAGT	Reverse complementary to TNV-A^{C} nt 2583-2609	Construction of Δ2610 with TN-435F based on pMTC27
TN-522R	TATTTTGCGATCGAAATGTGTT	Reverse complementary to TNV-A^{C} nt 2587-2608	Construction of Δ2609 based on pMTC27 with TN-523F
TN-523F	ACATGGCAGGAAAGAAGAACAACAACAATGGT	Corresponding to TNV-A^{C} nt 2610-2641	
TN-528R	CCATTGTGTTTCTTACCCTCACTACTC	Reverse complementary to TNV-A^{C} nt 2274-2300	Construction of Δ823+Δ2301~2581 based on TCPΔ823 with TN-529F
TN-529F	GACTCAACACATTTCGATCGCAAAATA	Corresponding to TNV-A^{C} nt 2582-2608	
TN-530F	CATTTCGATCGCAAAATAGACATGGCA	Corresponding to TNV-A^{C} nt 2591-2617	Construction of Δ823+Δ2301~2590 with TN-528R based on TCPΔ823
TN-531F	GCAAAATAGACATGGCAGGTTCTTGTA	Corresponding to TNV-A^{C} nt 2601-2619 and nt 3443-3450	Construction of Δ823+Δ2301~2600 with TN-528R based on TCPΔ823

TN-532F	GACATGGCAGGTTCTTGTACTGTAACT	Corresponding to TNV-A^{C} nt 2609-2619 and nt 3443-3458	Construction of Δ823+Δ2301~2608 with TN-528R based on TCPΔ823
AGUS-3	CAAACAATAGTTCTTGTACTGTAACTCG ACC	Corresponding to TNV-A^{C} nt 3443-3563, the underlined nucleotides are corresponding to the GUS ORF nt 1-23	Construction of pTGUS-I based on pMTC27 with AGUS-4, AGUS-5, AGUS-6, TN-132 and TN-64
AGUS-4	GTACAAGAACTATTGTTTGCCTCCCTGCT GC	Reverse complementary to the GUS ORF nt 1791-1812, the underlined nucleotides are complementary to TNV-A^{C} nt 3443-3451	
AGUS-5	GCAAAATAGACATGTTACGTCCTGTAGA AAC	corresponding to the GUS ORF nt 1-20, the underlined nucleotides are corresponding to TNV-A^{C} nt 2601-2611	
AGUS-6	GACGTAACATGTCTATTTTGCGATCGAA ATG	Reverse complementary to TNV-A^{C} nt 2591-2611, the underlined nucleotides are complementary to the GUS ORF nt 1-10	
AGUS-7	GAAGAACATGTTACGTCCTGTAGAAACC CC	corresponding to the GUS ORF nt 1-23, the underlined nucleotides are corresponding to TNV-A^{C} nt 2623-2629	Construction of pTGUS-II with AGUS-3, AGUS-4, AGUS-8, TN-132 and TN-64 based on pTGUS-I
AGUS-8	GTAACATGTTCTTCTTTCCTGCCATGTC	Reverse complementary to TNV-A^{C} nt 2609-2629, the underlined nucleotides are complementary to the GUS ORF nt 1-7	
AGUS-11	TTCTTCTTTCCTGCCATCTATTGTTTGCCT CCCTGCTGCGG	Reverse complementary to the GUS ORF nt 1789-1812, the underlined nucleotides are complementary to TNV-A^{C} nt 2612-2628	Construction of pTGUS-I 17 with AGUS-10, TN-132 and TN-64 based on pTGUS-I
AGUS-10	ATGGCAGGAAAGAAGAATTCTTGTACTG TAACTCGACCAATGCCT	Corresponding to TNV-A^{C} nt 2612-2628 (the underlined nucleotides) and nt 3443-3570	
AGUS-14	GGCAAACAATAGAATATCATCATTCTCCT AGTAC	Corresponding to TNV-A^{C} nt 2548-2569, the underlined nucleotides are corresponding to the GUS ORF nt 1801-1812	Construction of pTGUS-I 81 with AGUS-15, TN-132 and TN-64 based on pTGUS-I
AGUS-15	GATGATATTCTATTGTTTGCCTCCCTGCT GCGG	Reverse complementary to the GUS ORF nt 1789-1812, the underlined nucleotides are complementary to TNV-A^{C} nt 2548-2556	
AGUS-12	CAAACAATAGAGTGAAACATCTCAGAA ATCAC	Corresponding to TNV-A^{C} nt 2335-2356, the underlined nucleotides are corresponding to the GUS ORF nt 1803-1812	Construction of pTGUS-I 294 with AGUS-13, TN-132 and TN-64 based on pTGUS-I
AGUS-13	GATGTTTCACTCTATTGTTTGCCTCCCTG	Reverse complementary to the GUS ORF nt 1791-1812,	

	CTGC	the underlined nucleotides are complementary to TNV-A^C nt 2335-2345	
TN-487F	CCTACTTTGCAATTCTCATCCTCATAT	Corresponding to TNV-A^C nt 2488-2514	Construction of p2488~2629-3'UTR based on pTCPΔ814 with TN-563R
TN-563R	CTATAGTGAGTCGTATTACAATCGTCG	Reverse complementary to the sequence of pMTC27, the nucleotides for T7 promoter are underlined.	
TN-497F	CTAGTCTAGATTCTTGTACTGTAACTCGACC	Corresponding to TNV-A^C nt 3443-3464, *Xba*I and its terminal nucleotides are underlined.	Construction of p2488~2629-Luc-3'UTR based on p2488~2629-3'UTR with TN-498R
TN-498R	CATGCCATGGGTTCTTCTTTCCTGCCATGTCT	Reverse complementary to TNV-A^C nt 2608-2629, *Nco*I and its terminal nucleotides are underlined.	
TN-577F	CTAGTCTAGACCTACTTTGCAATTCTCA	Corresponding to TNV-A^C nt 2488-2505, *Xba*I and its terminal nucleotides are underlined.	Construction of pLuc-2488~2629-3'UTR based on p2488~2629-3'UTR with TN-573R
TN-573R	catgccatggCTATAGTGAGTCGTATTACAATCGTCG	Reverse complementary to the sequence of pMTC27, the nucleotides for T7 promoter are underlined, the small letters indicate *Nco*I and its terminal nucleotides.	
TN-561F	CCAACAATATCATCATTCTCCTAGTACT	Corresponding to TNV-A^C nt 2543-2570.	Construction of pGL-T87-Luc with TN-563R based on pGL-T142-Luc
TN-562F	GACATGGCAGGAAAGAAGAAC	Corresponding to TNV-A^C nt 2609-2629.	Construction of pGL-T21-Luc with TN-563R based on pGL-T142-Luc
Luc-14F	ATGGAAGACGCCAAAAACATAAAGAA	Corresponding to the ORF of firefly luciferase nt 1-26	Construction of pGL-Luc with TN-563R based on pGL-T142-Luc
Luc-15R	TTACACGGCGATCTTTCCGCC	Reverse complementary to the ORF of firefly luciferase nt 1633-1653	Construction of pGL-Luc-T87 with TN-561F based on pGL-Luc-T142； construction of pGL-Luc-T21 with TN-562F, based on pGL-Luc-T142.
TN-591F	*ctaagtatgcatggcattccggtactgttggtaaagccacc*ATGGAAGACGCCAAAAACATAAAGA	Corresponding to the ORF of firefly luciferase nt 1-25, the small letters indicate randomly selected nucleotides (RN) corresponding to the position nt 47-87 of pGL3-Basic	Construction of pGL-RN-Luc based on pGL-T142-Luc with TN-593R
TN-593R	gtcgcagatctcgagcccgcgctagcacgcgtaagagctcCTATAGTGAGTCGTATTACAATCGTC	Reverse complementary to the sequence of pGL-2488~2629-Luc, the nucleotides for T7 promoter are underlined, and the small letters indicate randomly selected nucleotides (RN) complementary to the position nt 7-46 of pGL3-Basic	
TN-578F	CCGGAATTCGACATGGCAGGAAAGAAGAAC	Corresponding to TNV-A^C nt 2609-2629, *Eco*RI and its terminal nucleotides are underlined.	Construction of p35S:T21-Luc with TN-581R based on pGL-T21-Luc

TN-579F	CCGGAATTCCAACAATATCATCATTCTCCTAGTAC	Corresponding to TNV-A^{C} nt 2543-2569, *Eco*RI and its terminal nucleotides are underlined	Construction of p35S:T87-Luc with TN-581R based on pGL-T87-Luc
TN-580F	CCGGAATTCCCTACTTTGCAATTCTCATC	Corresponding to TNV-A^{C} nt 2488-2507, *Eco*RI and its terminal nucleotides are underlined.	Construction of p35S:T142-Luc based on pGL-T142-Luc with TN-581R
TN-581R	CTAGTCTAGATTACACGGCGATCTTTCCGCC	Reverse complementary to the ORF of firefly luciferase nt 1633-1653, *Xba*I and its terminal nucleotides are underlined.	
Luc-16F	CCGGAATTCATGGAAGACGCCAAAAACATAAAGAA	Corresponding to the ORF of firefly luciferase nt 1-26, *EcoR*I and its terminal nucleotides are underlined.	Construction of p35S:Luc with TN-581R based on pGL-Luc
TN-584F	CCGGAATTCGAGCTCTTACGCGTGCTAGCG	Corresponding to RN in pGL-RN-LUC, *Eco*RI and its terminal nucleotides are underlined	Construction of p35S:RN-Luc with TN-581R based on pGL-RN-Luc
TN-570F	CATTCTAGAGTCGGGGCGGCCGCC	Corresponding to the sequence of pGL-Luc, *Xba*I and its terminal nucleotides are underlined	Construction of pGL-*EcoR*I:*Xba*I based on pGL-Luc with TN-571R
TN-571R	CCGGAATTCTATAGTGAGTCGTATTACAAT	Reverse complementary to the sequence of pGL-Luc, *Eco*RI and its terminal nucleotides are underlined	
TN-490R	CATGCCATGGTTTAGACTCTTTGGCTGAGT	Reverse complementary to TNV-A^{C} nt 40-59, *Nco*I and its terminal nucleotides are underlined	Construction of p5'UTR-*Nco*I:*Xba*I-3'UTR with TN-497F based on pTCPΔ814
TN-491F	CATGCCATGGCCAACAATATCATCATTCTCCTAGTAC	Corresponding to TNV-A^{C} nt 2543-2569, *Nco*I and its terminal nucleotides are underlined	Construction of pUTR-T87-Luc with TN-581R based on pGL-2543~2629-Luc$_{\Delta NcoI}$
TN-492F	CATGCCATGGGAGCTCTTACGCGTGCTAGCG	Corresponding to RN in pGL-RN-Luc, *Nco*I and its terminal nucleotides are underlined	Construction of pUTR-RN-Luc with TN-581R based on pGL-RN-Luc

a) The GenBank accession number of TNV-A^{C} is AY546104
